# Neurogenesis and cell cycle-reactivated neuronal death during pathogenic tau aggregation

**DOI:** 10.1111/j.1601-183X.2007.00377.x

**Published:** 2007-10-22

**Authors:** K Schindowski, K Belarbi, A Bretteville, K Ando, L Buée

**Affiliations:** † Inserm U837, Place de Verdun, Lille Cedex; ‡ Université Lille 2, Faculté de Médecine, Institut de Médecine Prédictive et Recherche Thérapeutique, rue Michel Polonowski Lille Cedex, France

**Keywords:** Alzheimer disease, AT8, AT100, cyclin B, cyclin D, doublecortin, NeuroD, TUC-4

## Abstract

The aim of the present study was to investigate the relation between neurogenesis, cell cycle reactivation and neuronal death during tau pathology in a novel tau transgenic mouse line THY-Tau22 with two frontotemporal dementia with parkinsonism linked to chromosome-17 mutations in a human tau isoform. This mouse displays all Alzheimer disease features of neurodegeneration and a broad timely resolution of tau pathology with hyperphosphorylation of tau at younger age (up to 6 months) and abnormal tau phosphorylation and tau aggregation in aged mice (by 10 months). Here, we present a follow-up of cell cycle markers with aging in control and transgenic mice from different ages. We show that there is an increased neurogenesis during tau hyperphosphorylation and cell cycle events during abnormal tau phosphorylation and tau aggregation preceding neuronal death and neurodegeneration. However, besides phosphorylation, other mechanisms including tau mutations and changes in tau expression and/or splicing may be also involved in these mechanisms of cell cycle reactivation. Altogether, these data suggest that cell cycle events in THY-Tau22 are resulting from neurogenesis in young animals and cell death in older ones. It suggests that neuronal cell death in such models is much more complex than believed.

Tau pathology is observed in several human neurodegenerative disorders such as Alzheimer’s disease (AD), Pick disease, frontotemporal dementia, corticobasal degeneration and progressive supranuclear palsy. In each of these disorders, called tauopathies, the accumulation of the abnormally hyperphosphorylated tau is associated with neurofibrillary degeneration and dementia. The discovery of mutations in the tau gene and their cosegregation with the disease in the inherited frontotemporal dementia with parkinsonism linked to chromosome-17 (FTDP-17) has established that abnormalities in tau protein as a primary event can lead to neurodegeneration and dementia (Hutton *et al.* 1998; Poorkaj *et al.* 1998; Spillantini *et al.* 1998). Interestingly, these tauopathies can be biochemically differentiated by their pattern of hyperphosphorylated isoforms, the so-called bar code (Buee *et al.* 2000; Sergeant *et al.* 2005). Hyperphosphorylation of tau precedes its accumulation into neurofibrillary tangle (NFT) in the affected neurons in AD (Sahara *et al.* 2002). Among the kinases involved in tau hyperphosphorylation, both cyclin-dependent kinase-5 (cdk5) and glycogen synthase kinase-beta (GSK3β) are of particular interest (Ahlijanian *et al.* 2000; Hamdane *et al.* 2003; Leroy *et al.* 2007; Patrick *et al.* 1999; Sengupta *et al.* 2006).

On one hand, cell cycle events were reported during neurofibrillary degeneration and neuronal death (Busser *et al.* 1998; Herrup & Busser 1995; Husseman *et al.* 2000; Yang *et al.* 2003). CDK5-p25-mediated hyperphosphorylation has been shown to be involved in reactivation of neuronal cell cycle followed by neuronal death (Hamdane & Buee 2007; Hamdane *et al.* 2005). On the other hand, cell cycle events are also encountered in neurogenesis. Adult hippocampal neurogenesis is not only involved in learning and the storage of memory (Kempermann 2002; Kempermann *et al.* 2002, 2004) but also in postlesional remodeling (Dempsey & Kalluri 2007; Gray *et al.* 2002). In addition, there is an increase in neurogenesis-related proteins in AD (Jin *et al.* 2004), but it is not clear if these changes are not related to proliferation of glial and vascular factors (Boekhoorn *et al.* 2006a).

The aim of the present study was to investigate the relation between neurogenesis, cell cycle reactivation and neuronal death during a sequential tau pathology. Lately, we generated a novel tau transgenic mouse line THY-Tau22 with two FTDP-17 mutations in a human tau isoform (Schindowski *et al.* 2006). This mouse displays the typical biochemical phosphorylation pattern of human tau in AD, NFT-like inclusions, neuropil threads (NT), paired haired filaments, ghost tangles, neuronal loss, astrogliosis, loss of functional synapes and impaired cognition. The THY-Tau22 mouse model displays a broad timely resolution of tau pathology with hyperphosphorylation of tau at younger age (up to 6 months) and abnormal tau phosphorylation and tau aggregation in aged mice (by 10 months).

Here, we present a follow-up of cell cycle markers with aging in control and transgenic mice. We show that there is an increased neurogenesis during tau hyperphosphorylation and cell cycle events during abnormal tau phosphorylation and tau aggregation preceding neuronal death and neurodegeneration.

## Material and methods

### Animals

THY-Tau22 mice were generated and characterized as recently described with a construct containing human tau46 mutated at G272V and P301S under the Thy1.2 promoter (Schindowski *et al.* 2006). As indicated in the original characterization work of the model, all transgenic THY-Tau22 mice used in the present study were heterozygous. Non-transgenic littermates were used as controls for all experiments. Animals were housed in small social groups in standard cages with free access to water and chow (rodent standard diet, Altromin, Lage, Germany) and a 12 hours light–dark cycle. Some mice were treated with 50 mg/kg 5-bromo-2′-deoxyuridine (BrdU) (Sigma-Aldrich, Lyon, France) intraperitoneal (i.p.) for 7 days and sacrificed 14 days later. Animals were either killed by cervical dislocation, brains were dissected and stored at −80 °C or sequentially perfused with 0.9% NaCl and 4% paraformaldehyde in phosphate-buffered saline, the dissected brains were postfixed overnight in 4% paraformaldehyde and either dehydrated for paraffin-embedding or cryopreserved in 30% sucrose for cryosections. All experiments on animals were performed following the approval of the Institute of Laboratory Animal Resources Committee, in accordance with standards for the care and use of laboratory animals and with French and European Community rules.

### Western blot analysis

Whole brains were dissected by separating the cortex from the hippocampus and thalamus. Hippocampus-enriched preparations were sonicated in Cell-Lysis Buffer (Cell Signaling Technology, Danvers, MA, USA) and then boiled at 100 °C for 10 minutes. Ten micrograms of total protein were resolved on sodium dodecyl sulphate–polyacrylamide gel electrophoresis, blotted onto nitrocellulose or polyvinylidene fluoride (PVDF) membranes (all from Invitrogen, Cergy Pontoise, France), incubated with appropriate antibodies (Table 1) and developed using the enhanced chemiluminescence kit (Amersham, Orsay, France). Protein levels were visualized and quantified using an imaging system (LAS-3000 2.0; Fuji Photo Film Co Ltd [Clichy, France]). For the detection of apoptosis, we used SH-SY5Y cells treated for 6 hours with 1 μM staurosporine (Sigma-Aldrich) as positive control and untreated SH-SY5Y as negative control (Delobel *et al.* 2003).

**Table 1:** Specificity, dilution and source of antibodies used in this study

Antibody	Species	Specificity or clone	Dilution	Source
AT8	Mouse	Tau; pSer202/pThr205	1:10 000	Innogenetics
AT100	Mouse	Tau; pThr212/pSer214	1:2000	Innogenetics
BrdU	Mouse	5-bromo-2′-deoxyuridine	1:10	Roche
β-tubulin	Rabbit	T3526	1:1000	Sigma
Caspase 3	Rabbit	Full caspase 3	1:1000	Cell Signaling
Cyclin B	Rabbit	M-20	1:1000	SantaCruz Biotechnology
Cyclin D	Rabbit	06-137	1:1000 (WB); 1:100 (IHC)	Upstate
Cyclin D1	Rabbit	H-295	1:100 (IHC)	SantaCruz Biotechnology
DCX	Goat	Doublecortin C-18	1:1000 (WB); 1:100 (IHC)	SantaCruz Biotechnology
NeuroD	Rabbit	H-76	1:1000 (WB); 1:100 (IHC)	SantaCruz Biotechnology
p27^KIP1^	Rabbit	C-19	1:1000 (WB); 1:100 (IHC)	SantaCruz Biotechnology
p21^CIP1^	Rabbit	C-19	1:250 (WB); 1:100 (IHC)	SantaCruz Biotechnology
TUC-4	Rabbit	ULIP1, CRMP4	1:5000	Chemicon

Innogenetics (Gent, Belgium); Roche (Mannheim, Germany); Sigma-Aldrich (Lyon, France); Cell Signalling Technologies (Danvers, MA, USA); SantaCruz Biotechnologies (Santa Cruz, CA, USA); Upstate/Millipore (Billerica, MA, USA); Chemicon (Limburg, Germany); WB, western blotting; IHC, immunohistochemistry.

### Immunohistochemistry and immunofluorescence

Cryosections were cut at 14 μm. 5-bromo-2′-deoxyuridine and TdT-mediated biotin–dUTP nick-end labeling (TUNEL) stainings (both from Boehringer Mannheim-Roche, Mannheim, Germany) were performed according to the manufacturer’s instructions. At 6 months of age, THY-Tau22 exhibited changes in doublecortin (DCX), which were significantly altered and therefore 6 months were also chosen for BrdU experiments. DNAse I-treated sections were used as positive controls for TUNEL staining. Primary antibodies (listed in Table 1) were incubated overnight at 4 °C. All secondary antibodies including biotinylated-, fluorescein- and Texas Red-coupled, ABC-kit and DAB/Ni were from Vector Laboratories (Burlingame, CA, USA). Hematoxylin staining (Sigma-Aldrich) was performed according to standard procedures. Paraffin sections (8 μm) were Gallyas silver-stained according to Braak & Braak (1991).

### Statistics

Data were quantified with NIH Image software, and statistics were analyzed by Student’s *t*-tests (Prism Graphpad, San Diego, CA, USA).

## Results

### Tau pathology in THY-Tau22 mice

**Figure 1:**
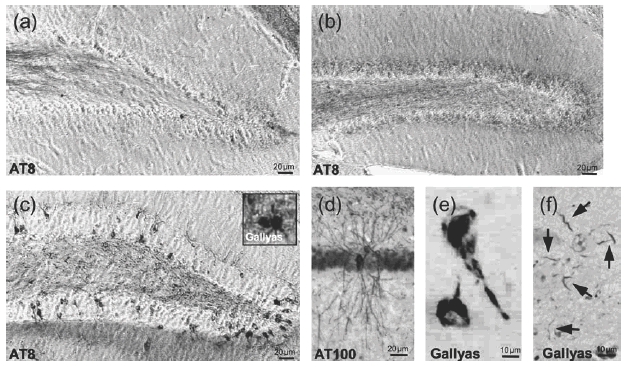
Tau pathology in aged THY-Tau22 (a) AT8-ir in the DG in a 3-month-old THY-Tau22 mice. Tau hyperphosphorylation is restricted to axons but absent in cell bodies. (b) AT8-ir in the DG in a 6-month-old THY-Tau22 mice. Mossy fibers and molecular layer show tau hyperphosphorylation. (c) AT8-ir in the DG in a 15-month-old THY-Tau22 mice with massive degeneration of AT8-positive fibers and extensive AT8-ir of cell bodies fibers. Rare Gallyas-positive stained NFT-like inclusions were found in DG at this age (inset). (d) AD-specific AT100-ir indicative for abnormal tau phosphorylation in CA1 pyramidal neurons in THY-Tau22 at 12 months. (e) NFT-like inclusions (Gallyas positive) were found numerously in the CA1 and subiculum of THY-Tau22 at 12 months. (f) In 12-month-old THY-Tau22 mice, NT (Gallyas positive) occurred frequently in the subiculum (arrows). Representative photos are shown.

In THY-Tau22 transgenic mice, brain sections exhibit hyperphosphorylation of tau [AT8-immunoreactivity (AT8-ir) tau phosphorylated at Ser202 and Thr205] and abnormal phosphorylation of tau [AT100-immunoreactivity (AT100-ir), tau phosphorylated at Thr212 and Ser214], NFT-like inclusions and NT (both Gallyas positive; Fig. 1) (Schindowski *et al.* 2006). In 3- to 6-month-old THY-Tau22 mice, tau hyperphosphorylation and NFTs are detected in the hippocampal formation and some cortical areas and their amount increases rapidly in aging (data not shown). The cell bodies of the dentate gyrus (DG) are relatively spared at young age, but their neurites show clearly AT8-ir (Fig. 1a,b). Tau phosphorylation and NFT-like inclusions were modest in the DG (Fig. 1c) but massively detected in pyramidal neurons of the CA1 subfield and the subiculum (Fig. 1d–f) in old animals.

### Neurodegeneration in aged THY-Tau22

To investigate the links between tau pathology and neuronal death, brain macroscopic and microscopic features were analyzed.

**Figure 2:**
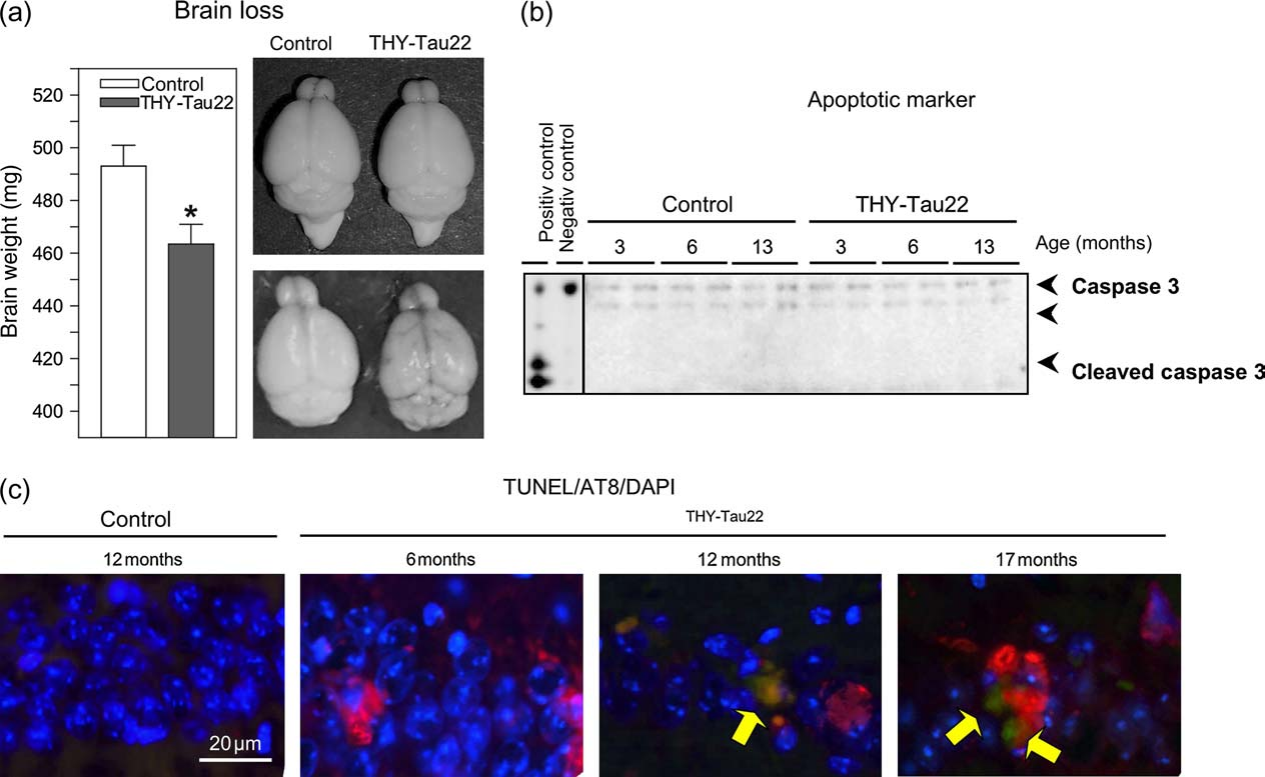
Neurodegeneration and apoptosis in old THY-Tau22 (a) Brain weight was significantly reduced (*P* < 0.014) in old THY-Tau22 mice compared with littermate controls (13–16 months) by 6%. Representative photographs of freshly prepared brains show a mild reduction in brain size. THY-Tau22: 463.4 ± 7.576 mg, *n* = 15; WT: 493.0 ± 8.025 mg, *n* = 12. (b) No cleavage of caspase 3 – as sign of apoptosis – was detectable in tau transgenic mice or in controls, indicating the absence of massive synchronized apoptotic cell death. Negative control, untreated growing SH-SY5Y cells; positive control, growing SH-SY5Y treated 1 μM staurosporine for 6 hours. *n* = 2 per genotype and age (c) TUNEL staining of the CA1-formation: TUNEL-positive cells (green, marked with arrows), AT8-positive cells (red) and the nuclear stain DAPI (blue) in control (12 months, *n* = 3) and THY-Tau22 mice at 6 months (*n* = 4), 12 months (*n* = 3) and 17 months (*n* = 1). Although TUNEL-positive cells were very rare, all of them colocalized with AT8-ir. The number of TUNEL-positive cells and AT8-ir increased in aged tau transgenic mice.*P < 0.05; WT, wildtype.

Brain mass and size of 13- to 16-month-old tau transgenic mice were mildly reduced by 6% compared with age- and sex-matched non-transgenic littermate controls (Fig. 2a). Lately, we had been shown a loss of pyramidal neurons in CA1 and subiculum by over 30% at this age (Schindowski *et al.* 2006). To investigate the presence of apoptosis in this model, we analyzed brain homogenates for caspase 3 cleavage (Fig. 2b). However, no active caspase 3 as sign for apoptotic cell death was found in brain tissues at any age in tau transgenic animals. Nevertheless, if apoptosis is not a main feature in this model, proteolytic products of caspase 3 may be diluted in brain homogenates. Thus, apoptosis markers were investigated at the neuronal level by histological procedures. Some TUNEL-positive pyramidal neurons were sometimes found in old THY-Tau22 mice in the CA1 subfield (Fig. 2c), while TUNEL staining was undetectable in control mice. Double staining with AT8 showed that apoptotic neurons were colocalized with phospho-tau (AT8-ir). Moreover, staining with the nuclear dye, DAPI, decreased from 12 months onwards in the CA1 region, indicating a progressive cell loss in THY-Tau22 mice. Therefore, apoptotic cell death seemed to be rather limited in our tau transgenic mouse, although the massive cell loss in CA1 and subiculum.

### Increase of cell cycle-related proteins during tau aggregation

It had been shown that cell cycle re-entry results in neuronal death in differentiated neurons (Greene *et al.* 2004), and several reports observe regulation of cell cycle-related proteins during the pathogenesis of AD (Andorfer *et al.* 2005; Busser *et al.* 1998; Herrup & Busser 1995). Recently, we showed tau pathology, neuronal death and reactivation of cell cycle in differentiated neuroblastoma cells overexpressing the CDK5 activator p25 (Hamdane *et al.* 2005). However, no change in p35 cleavage into p25 was observed in the THY-Tau22 model (data not shown).

**Figure 3:**
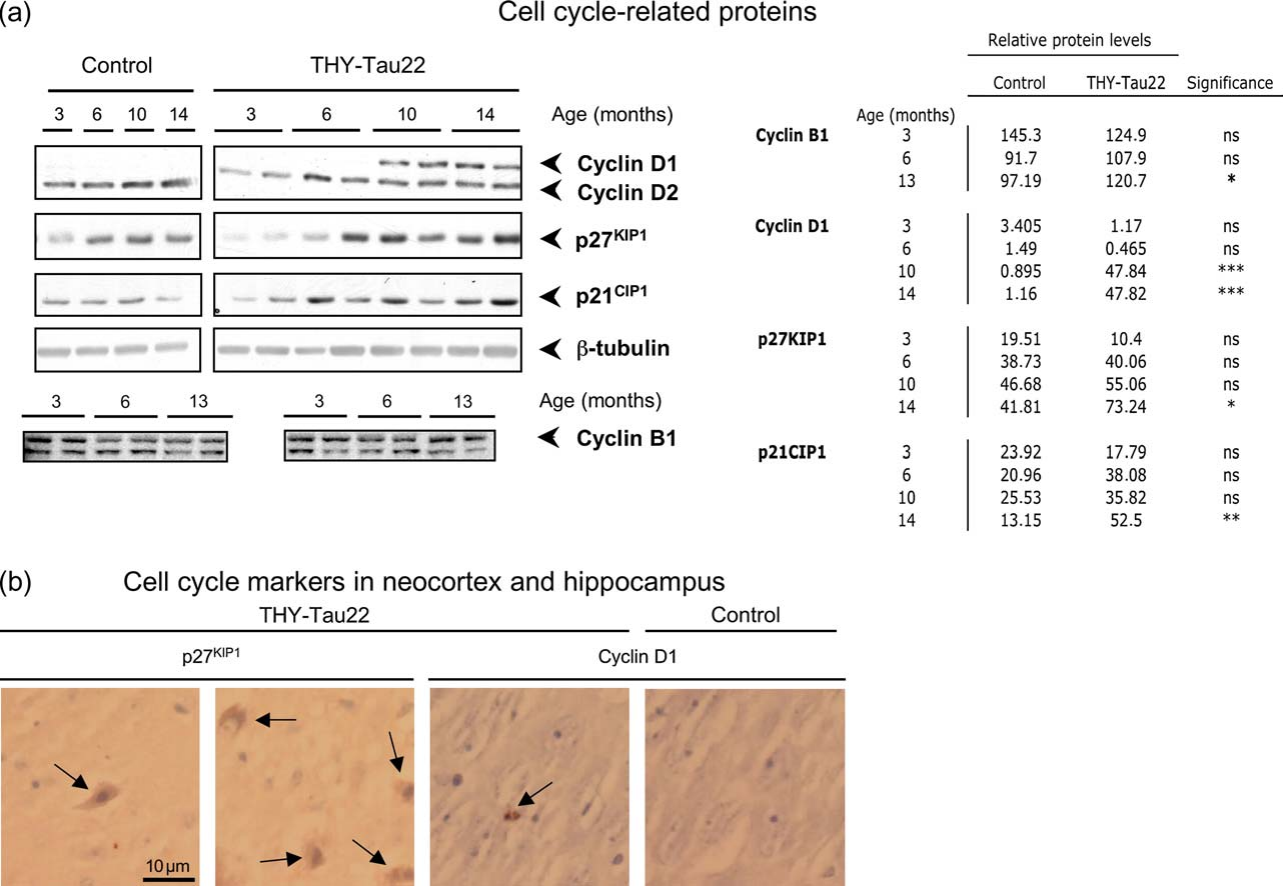
Upregulation of cell cycle-related proteins in old THY-Tau22 (a) Differential regulation of cell cycle-related proteins in hippocampal homogenates of THY-Tau22 and age-matched controls. Occurrence of cyclin D1 was the most notable change in tau transgenic mice, while protein levels of cyclin D2 did not change. p21^CIP1^ and p27^KIP1^ were also elevated in aged THY-Tau22. Cyclin B1 was mildly elevated. β-tubulin levels show that equal amounts of protein were loaded. Representative blots were shown; similar results were obtained with *n* = 3–6 mice per genotype and band intensities quantified. Mean optical densities are shown in the table. Significance was calculated with two-way analysis of variance and Bonferroni’s post-test. (b) In 13-month-old THY-Tau22 mice (*n* = 4), hippocampus and cortex stained for cell cycle markers cyclin D1 (CA1) and p27^KIP1^ (frontal cortex) were visualized with DAB (brown), counterstained with hematoxylin (blue) and similar field in an age-matched control mouse. Arrows indicate cells positive for the respective cell cycle marker. p27^KIP1^ was observed in controls and THY-Tau22, but the number of positive cells was increased in the latter. The cytoplasmic localization was identical. Cyclin D1 in nuclear localization was only found in old tau transgenics but not in age-matched controls.*P < 0.05; **P < 0.01; ***P < 0.001.

To link the effects of tau pathology caused by a FTDP-17 mutation on cell cycle events, major cell cycle-related proteins were investigated. Cyclin D1 is a protein that is required to leave the G0 phase and enter the G1 phase of the cell cycle and has been shown to be an early event leading to retinoblastoma (Rb) phosphorylation and the onset of neuronal death (Greene *et al.* 2004; Hamdane *et al.* 2005). In aged THY-Tau22 mice, during the stage when abnormal tau phosphorylation and tau aggregation occur, cyclin D1 was detected in brain homogenates, while it remained undetectable in young THY-Tau22 and control mice at any age (Fig. 3a). The hippocampal formation and cortical areas of tau transgenic animals contained cyclin D1-immunoreactive pyramidal neurons – with a nuclear localization (Fig. 3b). However, the total amount of detectable pyramidal neurons was rather low. Although several sections from non-transgenic age-matched control mice were analyzed, no cyclin D1 labeling was observed (Fig. 3b). The appearance of cyclin D1 is an early event in the normal cell cycle and a marker for G1 phase (for review, see Maller 1991; Nurse 1990; Pines 1993, 1995; Pines & Hunter 1994). In addition, levels of cyclin B1, the regulatory subunit of the cdc2-kinase, a relevant marker for G2 phase, were also increased in aged THY-Tau22 (Fig. 3a).

### Increased neurogenesis during tau hyperphosphorylation

Interestingly, p21^CIP1^ and p27^KIP1^, both inhibitors of the cyclin D-CDK4 complex (Gartel & Radhakrishnan 2005; Kwon *et al.* 1996), were also elevated in the tau transgenic mice (Fig. 3a,b). Interestingly and similar to what is described in AD (Busser *et al.* 1998), the localization of p27^KIP1^ was mainly cytoplasmic, indicating that it was not active (Fig. 3b).

**Figure 4:**
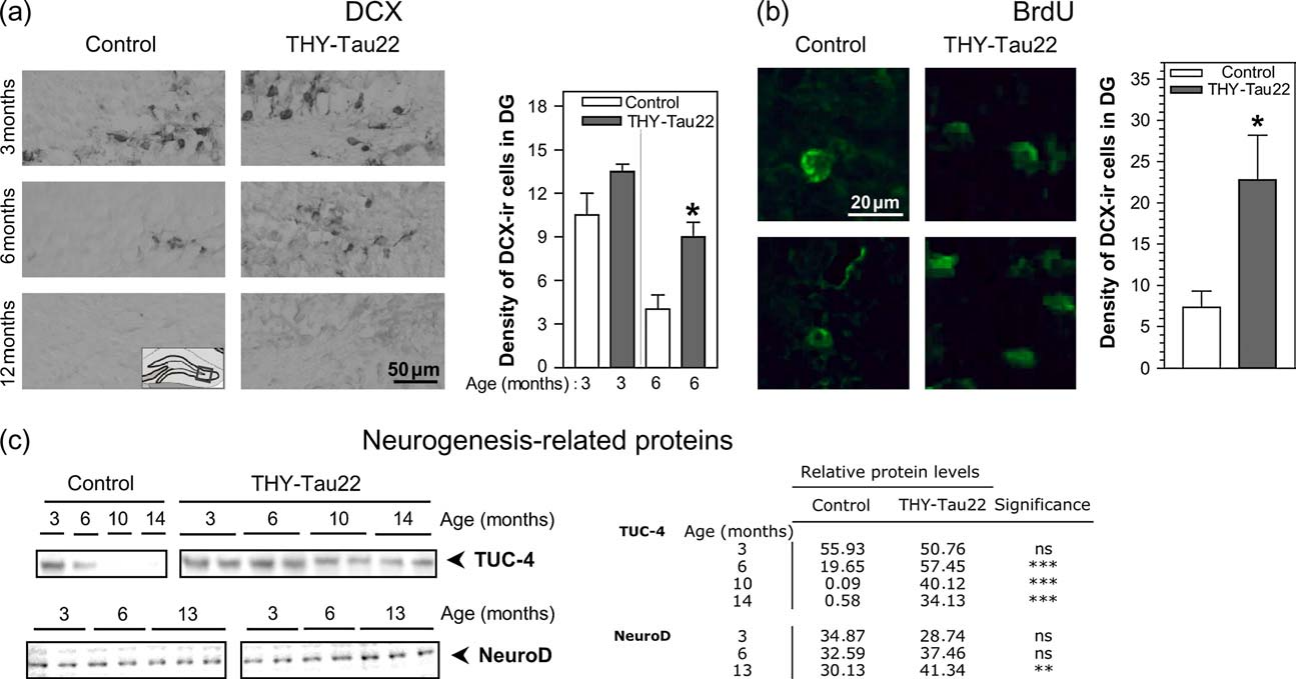
Increased and delayed neurogenesis in adult THY-Tau22 (a) DCX-ir in the DG of THY-Tau22 mice compared with WT at 3 months (*n* = 2), 6 months (*n* = 3 WT and *n* = 4 Thy-Tau22) and 12 months (*n* = 3). Representative images are shown. Note the age-related decay of DCX levels in control mice, while in THY-Tau22, increased numbers of DCX-ir cells were also observed at 6 months. Quantification of DCX-ir cells in the DG showed a significant increase at 6 months (*P < 0.05 at 6 months, *n* = 6 per genotype). Cell density is number of cells per field (photo taken with 10 × 10 magnification, approximately 190 × 100 μm). A coronal section (14 μm) showing the hippocampus around bregma −1.6 to −1.7 was used per animal. (b) 6-month-old mice were treated with BrdU i.p. for 7 days and sacrificed 14 days later. The number of BrdU-positive cells in the DG is significantly increased (*P < 0.05) in THY-Tau22 mice (*n* = 4) compared with littermate controls (*n* = 3). (c) Immunoblot analysis from hippocampal brain homogenates of proteins involved in neurogenesis and neuronal differentiation: TUC-4 is highly significantly and NeuroD mildly elevated in old THY-Tau22 mice compared with controls. Representative blots were shown and equal amounts loaded; similar results were obtained with *n* = 3–6 mice per genotype and band intensities quantified. Mean optical densities are shown in the table. Significance was calculated with two-way analysis of variance and Bonferroni’s post-test.

Because presence of cell cycle events may be related to neurogenesis, levels of DCX, TUC-4 and NeuroD, all being markers for neuronal differentiation (von Bohlen Und Halbach 2007), were analyzed in tau transgenic and control mice at different ages. First, DCX, a protein involved in neuronal differentiation and neurogenesis (Gleeson *et al.* 1998), was studied by immunohistochemistry. It was present in the DG of littermate control mice at 3 months but was significantly decreased at 6 months and undetectable by 12 months (Fig. 4a). The DCX-immunoreactivity (DCX-ir) in the DG of THY-Tau22 mice was increased at 3 months, and its decrease with age was delayed. At 6 months, the DCX levels were 2.2-fold higher than in the controls (Fig. 4a). Moreover, TUC-4 (also known as ULIP1 or CRMP4), a protein that is involved in neuronal maturation and differentiation (Cameron & McKay 2001), was also present at 3 months in both control and THY-Tau22 mice. In control mice, the levels of TUC-4 were decreased at 6 months and almost undetectable at 10–14 months, whereas surprisingly, the levels of TUC4 remained elevated in THY-Tau22 mice at 10 and 14 months (Fig. 4c).

NeuroD is a basic helix-loop-helix transcription factor and is expressed at the time of the terminal differentiation into mature neurons (Lee *et al.* 1995). The levels of NeuroD were mildly increased in aged THY-Tau22 compared with age-matched littermate controls (Fig. 4c).

To determine if these increased levels of neurogenesis-related protein resulted also in increased amounts of newborn cells, 6-month-old mice were treated with the base-analog BrdU during 7 days and sacrificed 14 days later. The amount of BrdU-positive cells was nearly three times higher in the granule DG cell layer of THY-Tau22 mice compared with controls, indicating that the increase of *de novo* DNA synthesis resulted in viable cells (Fig. 4b). 5-bromo-2′-deoxyuridine staining was also observed in the subventricular zone and exceptionally in other brain areas.

## Discussion

Tau hyperphosphorylation and tau aggregation appear to have differential effects on differentiation and cell death. While more and more reports propose a protective role of tau hyperphosphorylation (Hamdane *et al.* 2005; Lee *et al.* 2005), tau aggregation is supposed to contribute to neuronal death (Ramsden *et al.* 2005). Hyperphosphorylation had been shown to be reversible, while tau aggregation is not (Santacruz *et al.* 2005).

The aim of this study was to link tau pathology to cell cycle events and differentiation subsequent to neurogenesis.

In AD brains, there is an increase in proteins that are involved in neuronal differentiation and maturation (Jin *et al.* 2004; Yang *et al.* 2003). An important finding of this report was the presence of these proteins in young tau transgenic mice. The major site of adult neurogenesis, besides the subventricular zone, is the DG. It should be emphasized that at the age of 3–6 months, fibers of the granular cell layer of the DG were immunoreactive for tau phosphorylation, but their cell bodies were relatively spared from tau pathology. Abnormal phosphorylated tau (AT100) and NFTs were mostly absent at this age (Schindowski *et al.* 2006). Therefore, early molecular events, likely involved in the regulation of neurogenesis and plasticity, were detected even before the onset of somatic hyperphosphorylated tau deposits (Boekhoorn *et al.* 2006b).

From our data, it is difficult to conclude that these changes are only related to tau phosphorylation. On one hand, tau expression and/or mutation may also have an impact on neuronal physiology, such as maturation and splicing. For instance, it was described that 4-repeat tau isoforms regulate hippocampal neurogenesis and promote neuronal differentiation (Sennvik *et al.* 2007). Conversely, the neonatal 3-repeat isoform is present in proliferating progenitor cells and associated with DCX expression and BrdU incorporation during adult neurogenesis in the rat hippocampus (Bullmann *et al.* 2007; Mosch *et al.* 2007). TUC-4 regulation is observed in tau-positive stem cells during neuronal differentiation (Munoz-Elias *et al.* 2003). Moreover, TUC-4 is also involved in F-actin bundling (Rooslenbroich *et al.* 2005). Recent data from experimental models of tauopathies suggest some relationships between actin-binding proteins and tau pathology (Blard *et al.* 2007; Fulga *et al.* 2007). Thus, some links may exist between TUC-4 and tau pathology through actin network and increased phase of neuronal differentiation. Nevertheless, more work is needed to find the exact relationships. On the other hand, we cannot exclude that neurogenesis, an event that is predominantly present in young adults, is dependent on factors that are mainly present in young brains, and it should be emphasized that in the present model, tau hyperphosphorylation correlates with an increased neurogenesis. Nevertheless, it had been shown that even aged brains are able to increase neurogenesis in response to an environmental enrichment (Kempermann *et al.* 2002) or cerebral lesions (Dempsey & Kalluri 2007; Gray *et al.* 2002; Kokaia & Lindvall 2003). Neurogenesis-related proteins have also been observed in mice expressing non-mutant human tau (Andorfer *et al.* 2005) and tau mutations (Kuhn *et al.* 2007).

**Figure 5:**
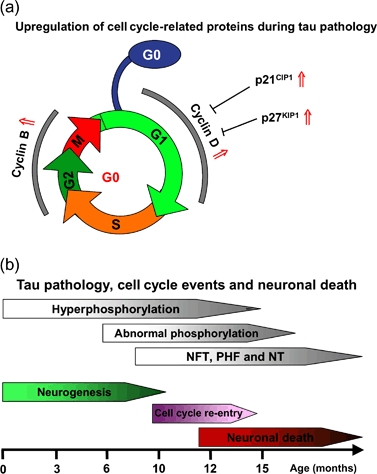
Cell cycle related events during tau pathology (a) Summary of observed cell cycle events in aged tau transgenic mice: Regulation of cyclin D during the G1 phase and cyclin B during G2 to mitosis transition. p21^CIP1^ and p27^KIP1^ inhibit the cyclin D/CDK4 complex. Changes in the THY-Tau22 mouse model are indicated with red arrows. indicates increase in proteins levels. (b) Summary of cell cycle events and neuronal death during different stages of tau pathology in THY-Tau22.

By contrast, the CA1 subfield, which is the brain region with the highest density of tau phosphorylation and NFT load in aged THY-Tau22 mice, shows later massive neurodegeneration and cell loss. Cyclin D1, a marker of neuronal cell death that is elevated in AD brain (Busser *et al.* 1998), was significantly increased by 10 months when tau phosphorylation and the formation of NFT are quite advanced in THY-Tau22. Interestingly, cyclin D1 is a key molecule of neurons leaving G0 and entering G1 (Greene *et al.* 2004). p21^CIP1^ and p27^KIP1^ are related proteins that inhibit cell cycle progression by interacting with cyclin D-CDK4 complex in the nucleus (el-Deiry *et al.* 1993; Gu *et al.* 1993; Harper *et al.* 1993; Polyak *et al.* 1994; Xiong *et al.* 1993). Moreover, p27 is essential for the translocation of cyclin D from the cytosol to the nucleus and is increased in tau models (Delobel *et al.* 2006) and AD brain and colocalized with NFT (Ogawa *et al.* 2003). The increase in p21 that is consistent with findings from different tau models (Delobel *et al.* 2006) and AD patients (Zhu *et al.* 2004). Figure 5a shows an overview of the regulation of cell cycle-related proteins during tau pathology: markers of G1 and G2 were significantly elevated during the stage of abnormal tau phosphorylation and tau aggregation and preceded neuronal loss.

It was suggested that failure in synaptic plasticity may lead to cell cycle dysfunction (Arendt & Bruckner 2007). However, cell cycle events had been linked so far to Aβ toxicity in AD (Copani *et al.* 2002). Here, reactivation of cell cycle and neuronal death are described in a model of tauopathy, emphasizing the impact of tau pathology on neuronal cell cycle re-entry. Moreover, phosphorylation of the cell cycle protein Rb triggered by the tau kinase p25/Cdk5 and activation of E2F-responsive genes occur in neuronal death (Hamdane *et al.* 2005). Excitingly, here, we detected neuronal cell cycle re-entry during the stage of abnormal tau phosphorylation and tau aggregation but preceding neuronal death.

Cell death and neuronal loss (Schindowski *et al.* 2006) were detected by 12 months or later in our mouse model. Therefore, the re-entry of cell cycle is preceding neuronal death in THY-Tau22 as we have shown recently in a cell model (Hamdane & Buee 2007). A short summary with time lines for tau pathology, cell cycle events and neurodegeneration of our mouse model is sketched in Fig. 5b. Neurodegeneration and cell loss have also been observed in other tau transgenic mouse models with tau mutations at sites P301S (Allen *et al.* 2002), P301L (Gotz *et al.* 2001; Lewis *et al.* 2000), V337M (Tanemura *et al.* 2002) and R406W (Ikeda *et al.* 2005; Lim *et al.* 2001; Zhang *et al.* 2004). However, evidence of apoptosis was only observed in P301L-mutated tau mice (Gotz *et al.* 2001; Santacruz *et al.* 2005) and in mice expressing non-mutated human tau (Andorfer *et al.* 2005).

Consistent with findings of AD brain material and tau transgenic mice (Colurso *et al.* 2003), we observed few TUNEL-positive cells in THY-Tau22 mouse brain, although the number of TUNEL-positive cells was significantly increased compared with age-matched controls. The levels of cleaved caspase 3 remained undetectable, but it should be taken into consideration that cleavage of caspases is a rather short event, and therefore, their detection is difficult in a dynamic not-synchronous system such as the brain. Excitingly, cytoplasmic p21^CIP1^ has been reported to prevent apoptosis by inhibiting activation of caspase 3 (Asada *et al.* 1999; Suzuki *et al.* 1998). Our data are comparable with reports about the absence of active caspase 3 in AD brain and tau mouse models (Andorfer *et al.* 2005).

Altogether, these data suggest that cell cycle events in THY-Tau22 are resulting from neurogenesis in young animals and cell death in older ones. It suggests that neuronal cell death in such models is much more complex than believed.
